# Effect of Enhanced Recovery After Surgery on Postoperative Recovery and Quality of Life in Patients Undergoing Laparoscopic Partial Nephrectomy

**DOI:** 10.3389/fonc.2020.513874

**Published:** 2020-10-14

**Authors:** Chenkui Miao, Aimei Yu, Han Yuan, Min Gu, Zengjun Wang

**Affiliations:** ^1^Department of Urology, The First Affiliated Hospital of Nanjing Medical University, Nanjing, China; ^2^Division of Urology, Department of Surgery, Brigham and Women’s Hospital, Harvard Medical School, Boston, MA, United States; ^3^Center for Quantitative Medicine, Duke-NUS Medical School, National University of Singapore, Singapore, Singapore; ^4^Department of Epidemiology, Harvard T.H. Chan School of Public Health, Boston, MA, United States; ^5^Department of Urology, The Second Affiliated Hospital of Nanjing Medical University, Nanjing, China

**Keywords:** enhanced recovery after surgery, laparoscopy, partial nephrectomy, kidney cancer, quality of life

## Abstract

Patients who underwent laparoscopic partial nephrectomy from the First Affiliated Hospital of Nanjing Medical University from May 2016 to May 2019 were randomly divided into enhanced recovery after surgery (ERAS) and control groups. The clinical indicators, preoperative and postoperative anxiety, depression, and postoperative quality of life were compared between the two groups. The recovery time, hospitalization cost, incidence of complications, and postoperative anxiety of patients in the ERAS group were lower than those of the control group. The satisfaction during hospitalization, scores of physical function, role function, emotional function, and general health status of the ERAS group were also significantly increased. Applying the ERAS to patients undergoing laparoscopic partial nephrectomy can improve their prognosis, experience of medical treatment, and life quality after surgery as well as have certain economic advantages.

## Introduction

With the advancement of medical treatment level and the transformation of people’s health concept, modern medicine has changed from a disease-centered biomedical model to a human-centered bio-psychological–social medical model (1). Enhanced recovery after surgery (ERAS) is a multidisciplinary treatment that meets the requirements of modern medicine, takes a series of interventions to help patients shorten their recovery time, reduce complications, and effectively improve their prognosis. Therefore, under the background of the modern medical model, the ERAS model is treasured by the health administrative departments and medical institutions. ERAS has been widely used in urology, including laparoscopic radical nephrectomy (2), radical cystectomy (3), adrenalectomy (4), partial nephrectomy (5), prostatic hyperplasia surgery (6), and robot-assisted laparoscopic radical prostatectomy (7), *etc*., achieving good clinical results. However, there are few studies on the implementation of ERAS in laparoscopic partial nephrectomy. This study takes the application of ERAS in laparoscopic partial nephrectomy as an example, investigating hospitalization days, hospitalization expenses, preoperative and postoperative anxiety and depression, patient satisfaction, and postoperative quality of life, and combines clinical efficacy data to analyze and comprehensively evaluates ERAS, providing a reference for policy-making of the health administrative department.

## Materials and Methods

### Patients and Randomization Methods

Approval for this study was granted by the Ethics Committee of the First Affiliated Hospital of Nanjing Medical University, and informed written consent was received from all participants (information not shown due to confidentiality principle).

In this study, we utilized kidney cancer patients who were admitted to the First Affiliated Hospital of Nanjing Medical University and underwent laparoscopic partial nephrectomy as the research object. The SAS9.4 software was used to generate 1:1 randomization. Patients were randomly divided into two groups based on randomization: one group was the implementation group of accelerated rehabilitation surgery (ERAS), and the other group was the control group with traditional treatment methods. A total of 240 patients were enrolled in this study, and 27 patients were lost to follow-up or dropped out. A total of 213 patients were followed up, including 110 in the ERAS implementation group and 103 in the control group.

### General Information

Renal cancer patients undergoing laparoscopic partial nephrectomy in the First Affiliated Hospital of Nanjing Medical University from May 2017 to May 2019 were randomly divided into the ERAS group and the control group, by using the SAS9.4 software. A total of 240 patients were enrolled in the present study. 27 patients were excluded for losing contact. A total of 213 patients were followed up, including 110 in the ERAS group and 103 in the control group. The inclusion criteria were as follows: (1) clinical diagnosis of primary renal cancer T1a (tumor maximum diameter ≤4 cm), no tumor metastasis; (2) laparoscopic partial nephrectomies; and (3) patients who were informed and consented to participate. The exclusion criteria of the study were as follows: (1) combined with other urinary system diseases; (2) serious cardiovascular and cerebrovascular diseases and respiratory diseases; (3) with anesthesia, surgical contraindications; and (4) postoperative pathological changes. The patients and their families were informed about our research in detail, and they agreed and signed the informed consent form. The ethics committee of the hospital approved this research.

### Perioperative Management Methods

Based on the routine management strategies of our center, we performed the traditional perioperative management program in the control group, while we used an optimized perioperative management plan in the ERAS group, such as preoperative mission to accelerate the rehabilitation program, preoperative fasting (6 h before surgery), preoperative water deprivation (2 h before surgery), taking 5% glucose 500 ml for 2 h before surgery, adjustment of drinking water for diabetic patients, no enema, keeping intraoperative warming, control of fluid input, postoperative active appropriate analgesia, postoperative fluid diet (6 h after surgery), postoperative normal diet (2 days after surgery), encouragement of early activity, postoperative removal of the catheter (2 days after surgery), and removal of the drainage tube (3 days after surgery). The intervention details are illustrated in Table 1.

**TABLE 1:** Perioperative management methods for the two groups of patients.

Perioperative management methods	Control group	ERAS group
Education	Routine preoperative medical knowledge related education	The content of preoperative education is more comprehensive, including an accelerated rehabilitation plan, which covers the approximate time of each stage of rehabilitation and various suggestions for promoting rehabilitation such as early eating and early activities
Preoperative fasting and preoperative water deprivation	Preoperative fasting (12 h before surgery), preoperative water deprivation (4 h before surgery)	Preoperative fasting (6 h before surgery), preoperative water deprivation (2 h before surgery), taking 5% Glucose 500 mL for 2 h before surgery, adjustment to drinking water for diabetic patients
Bowel preparation	Clean the enema with soap and water before surgery	No enema
Urinary catheter management	Extraction 3–5 days after surgery	Extraction 1–2 days after surgery
Drainage tube management	Extraction 5–6 days after surgery	Extraction 2–3 days after surgery
Intraoperative insulation	Not emphasized	Control indoor temperature, reduce unnecessary exposure of patients, use insulation blankets as appropriate; heat intravenous infusion water
Postoperative analgesia	Postoperative on-demand analgesia	Local anesthetic incision infiltration is used, and injected at different levels of tissues before the skin is sutured. After the operation, the intravenous pump is routinely placed and non-steroidal analgesics are given for active and appropriate analgesia.
Postoperative anticoagulation	Not emphasized	When drainage conditions permit, give low molecular weight heparin early for anticoagulation to prevent thrombosis
Liquid input	During the operation, 1500 ml of colloid and other liquids are routinely given, and 2500–3000 ml is routinely used for 3–4 days after the operation.	During the operation, coordinate with the anesthesiologist to control the administration of 500 ml of fluid as much as possible, and control the amount of fluid to be 1500 ml on the day after the operation, and then decrease the amount daily as the food intake increases.
Postoperative activities	Absolutely stay in bed for about 1 week after operation	Encouragement of early activity, postoperative removal of the catheter (2 days after surgery)
Postoperative eating	Water and fasting after operation, proper drinking after exhausting, and gradually transition to liquid, semi-liquid, general food	Postoperative fluid diet (6 h after surgery), postoperative 60–70% normal diet (2 days after surgery)

### Research Variables and Data Collection

#### Research Variables

Basic information, such as gender, age, weight, tumor side, tumor size, operation time, and amount of intraoperative bleeding, *etc*., were obtained from the patients. The following data were also collected and analyzed—clinical indicators: recovery time of bowel sounds, first exhaust time, first defecation time, removal time of catheter, and removal time of drainage tube; efficiency indicators: preoperative hospital stay time, postoperative hospital stay time, and total hospital stay time; benefit index: hospitalization cost; safety indicators: complication rate; susceptibility indicators: pre- and postoperative level of patients’ anxiety and depression, patient satisfaction, postoperative quality of life, *etc*.

#### Data Collection

The Self-Rating Anxiety Scale (SAS) and the Self-Rating Depression Scale (SDS) were filled out at the time of admission and discharge to obtain preoperative and postoperative anxiety and depression data. The four options of the SAS and the SDS correspond to four scores, respectively. The positive scores are scored by 1, 2, 3, and 4, and the reverse scores are 4, 3, 2, and 1. The total score is obtained through the sum of each question score, and the total score is multiplied by 1.25 to get the standard score. The higher the standard score, the more serious the symptoms. Patients fill out the satisfaction survey form at the time of discharge to obtain the overall satisfaction of the patient (1 point for bad, 2 points for ok, 3 points for good, 4 points for satisfied, and 5 points for perfect). At 3 months follow-up, the European Cancer Therapy Research Organization (EORTC) Quality of Life Measurement Scale [QLQ-C30 (V3.0)] was completed by patients to obtain postoperative quality of life. The quality of life measurement scale calculates the rough points according to different dimensions and normalizes the rough points: the total health status and symptom dimension is standardized as SS = [(RS - 1)/*R*] × 100, and the functional dimension is standardized as SS = [1 - (RS - 1)/*R*] × 100 (*R* stands for the full distance score of each dimension, RS stands for the rough points, and SS stands for the standardized score). The higher the functional dimension and the overall health status score are, the better the functional status and quality of life. The higher the symptom dimension score is, indicating more symptoms or problems, the worse the quality of life. We obtained demographic sociological data (such as gender, age, *etc.*), length of hospital stay, and hospitalization expenses, *etc.*, as well as intraoperative and postoperative data such as the amount of intraoperative bleeding, recovery time of bowel sounds, and complication rate, *etc.* from the medical case system and electronic medical record system.

### Statistical Analysis and Quality Control

The data was dual-track transferred using the EpiData 3.1 software, and the data was statistically analyzed using SPSS 16.0 software. The measurement data were described by mean ± standard deviation (*x* ± *s*) and analyzed by *t* test. The enumeration data is described as percentage, and the two groups are compared using chi-square tests or calibration chi-square tests or Fisher’s exact tests. *P* ≤ 0.05 was considered as statistically significant.

We controlled the research quality based on the following standards: (1) trained physicians are responsible for the selection of research subjects and performing inclusion and exclusion according to criteria; (2) ERAS has a set of standardized implementation paths, which should be strictly followed by the medical staff; (3) conduct uniform training for investigators before the investigation; and (4) enter the survey data through two tracks, verify data using EpiData, and utilize SPSS to perform statistical analysis.

## Results

### Baseline Characteristics of Two Groups

There were no statistically significant differences in gender, age, weight, tumor side, tumor size, and other general data between the two groups. The operation was successfully completed in both groups. There was no statistically significant difference in the operation time and intraoperative amount of bleeding between the two groups (Table 2). The two sets of data are balanced and comparable.

**TABLE 2:** Baseline information of the two groups of patients.

Category	ERAS group (*n* = 110)	Control group (*n* = 103)	χ^∧^2/*t* value	*P* value
Sex (men/women)	81/29	74/29	0.086	0.769
Age (years)	053.41 ± 14.25	055.63 ± 13.79	−1.155	0.249
Weight (kg)	66.64 ± 8.81	66.69 ± 7.72	−0.047	0.963
Tumor side (cases)			−0.006	0.936
Left	56	53		
Right	54	50		
Tumor diameter (cm)	02.56 ± 0.84	02.50 ± 0.90	−0.534	0.594
Operation time (minutes)	88.06 ± 5.43	87.82 ± 5.26	−0.338	0.736
Intraoperative amount of bleeding (mL)	241.22 ± 78.28	244.08 ± 85.63	−0.255	0.799

### Clinical Features and Safety Evaluation

All patients from both groups were treated properly and ultimately recovered well. The *t* test analysis of the two groups of data showed that the recovery time of bowel sounds, the time of first defecation, the time of removal of the catheter, and the time of removal of the drainage tube in the ERAS group were less than those in the control group (*P* < 0.001). There was no significant difference in the first exhaust time (*P* = 0.371). The details are presented in Table 3.

**TABLE 3:** Comparison of clinical effect between the two groups.

Category	Bowel sound recovery time (hours)	First exhaust time (hours)	First defecation time (hours)	Removal time of catheter (days)	Removal time of drainage tubes (days)
ERAS group (*n* = 110)	21.06 ± 2.32	35.08 ± 3.85	52.13 ± 7.47	1.99 ± 0.80	2.95 ± 0.83
Control group (*n* = 103)	29.34 ± 2.29	35.71 ± 6.03	72.93 ± 7.59	3.60 ± 1.11	4.62 ± 1.09
*t* Value	−26.156	−0.898	−20.159	−12.071	−12.577
*P* Value	<0.001	−0.371	<0.001	<0.001	<0.001

After different rehabilitation treatments, the two groups of patients improved significantly and were discharged without death. The incidence of complications in each group was recorded. The control group and the ERAS group consisted of 41 cases and 8 cases, respectively. The chi-square test shows that the incidence of complications in the ERAS group was lower than that in the control group (*P* < 0.001). The details are presented in Table 4.

**TABLE 4:** Postoperative complications in the two groups of patients.

Category	Preoperative low blood sugar	Postoperative shiver and high fever	Postoperative hypostatic pneumonia	Postoperative abdominal distention	Postoperative Stress ulcer	Postoperative cardiovascular and cerebrovascular accidents	Incision liquefaction	Urinary tract infection	Venous thrombosis	Total
ERAS group (*n* = 110)	1	1	0	4	1	0	0	1	0	8
Control group (*n* = 103)	3	8	3	7	5	2	4	5	4	41
χ^∧^2/χc^∧^2	0.327	4.603	1.491	1.084	1.755	/	2.501	1.755	2.501	31.785
*P* value	0.568	0.032	0.222	0.298	0.185	0.233	0.114	0.185	0.114	<0.001

### Efficiency and Benefit Evaluation

The average length of hospital stay was not statistically different (*t* = 1.743, *P* = 0.083) between the ERAS group [(1.85 ± 0.80) days] and the control group [(2.04 ± 0.82) days]. However, the average length of hospital stays in the ERAS group [(4.47 ± 1.11) days] is less than that in the control group [(7.44 ± 1.72) days], where the difference was statistically significant (*t* = 14.825, *P* < 0.001), and the total average hospital stay in the ERAS group [(6.32 ± 1.45) days] is less than that in the control group [(9.48 ± 1.89) days], where the difference was statistically significant (*t* = 13.612, *P* < 0.001).

Furthermore, the average hospitalization cost in the ERAS group was (4.55 ± 0.63) yuan, whereas the average hospitalization cost in the control group was (5.53 ± 0.61) yuan. By *t* test analysis, the hospitalization cost of the ERAS group was lower than that of the control group, and the difference was statistically significant (*t* = 11.560, *P* < 0.001).

### Emotional Outcomes Between Groups

Generally, there was no significant difference between the two groups of patients (*P* > 0.05) in terms of preoperative anxiety, depression, and overall satisfaction. The postoperative anxiety degree was lower in the ERAS group than in the control group (*P* < 0.001). The postoperative anxiety degree of the two groups was lower than that before the operation. There was no significant difference in preoperative and postoperative depression between the two groups (*P* > 0.05). The postoperative depression in both groups was lower than that before surgery. The hospital satisfaction score was higher in the ERAS group than in the control group (*P* < 0.001). More details are shown in Table 5.

**TABLE 5:** Preoperative and postoperative anxiety, depression, and overall satisfaction scores of the two groups.

Catergory	SAS anxiety score		SDS depression score		Overall satisfaction score
	Preoperative	Postoperative	Preoperative	Postoperative	
Control group	60.01 ± 11.28	50.84 ± 11.19	61.82 ± 7.00	47.63 ± 6.46	2.52 ± 0.93
ERAS group	61.90 ± 8.60	42.11 ± 8.56	61.58 ± 7.04	46.55 ± 6.59	4.12 ± 0.80
*t* Value	−1.366	6.359	0.25	1.215	−13.404
*P* Value	0.174	<0.001	0.803	0.226	<0.001

To reveal the postoperative quality of life, statistical analysis was performed on the standardized scores of different dimensions of the two groups. The results showed that somatic function, role function, emotional function, and general health status of the ERAS group were significantly higher than those of the control group (*P* < 0.001), but there was no statistical difference in cognitive function, social function, and symptom dimensions, including fatigue, nausea and vomiting, pain, shortness of breath, insomnia, loss of appetite, constipation, diarrhea, and financial difficulties (*P* > 0.05). Detailed data are presented in Table 6.

**TABLE 6:** Comparison of postoperative quality of life between the two groups.

Dimensions of life quality	ERAS group	Control group	*t* value	*P* value
**Functional dimensions**				
Somatic function	86.30 ± 7.04	73.20 ± 14.46	–8.316	<0.001
Role function	82.73 ± 12.76	71.68 ± 11.63	–6.589	<0.001
Emotional function	76.06 ± 8.97	68.85 ± 8.57	–5.99	<0.001
Cognitive function	92.12 ± 8.36	91.59 ± 8.69	–0.458	0.647
Social function	79.24 ± 10.39	77.83 ± 9.73	–1.021	0.308
General health	76.59 ± 9.07	67.23 ± 10.32	–7.041	<0.001
**Symptom dimensions**				
Fatigue	25.05 ± 8.57	26.11 ± 7.58	0.949	0.343
Nausea and vomiting	6.52 ± 8.77	7.12 ± 8.92	0.499	0.619
Pain	15.76 ± 9.26	16.67 ± 9.62	0.702	0.483
Shortness of breath	8.48 ± 14.59	8.74 ± 14.73	0.126	0.9
Insomnia	18.48 ± 16.64	18.45 ± 17.29	–0.016	0.987
Loss of appetite	12.12 ± 16.11	13.92 ± 16.52	0.803	0.423
Constipation	12.42 ± 17.98	13.27 ± 18.28	0.34	0.734
Diarrhea	11.52 ± 17.16	11.65 ± 17.28	0.057	0.954
Financial difficulties	32.73 ± 26.32	33.33 ± 26.81	0.166	0.868

## Discussion

Enhanced recovery after surgery is derived from fast-track surgery (FTS) and is widely used in clinical practice currently. Essentially, it is a multidisciplinary comprehensive treatment method, through a series of interventions, optimizing the perioperative treatment plan and reducing the physiological and psychological traumatic stress of patients, in order to improve the patient’s prognosis and shorten the length of hospital stay (8). The core of the ERAS concept is patient-centered, and the treatment plan has individualized features, all of which are based on humanity (9). Renal cell carcinoma ranks seventh in the incidence of cancer in men and 10th in women (10). Radical nephrectomy is a classic surgical procedure in traditional laparoscopic treatment of renal cancer. At present, results of worldwide research show that if tumor size does not exceed 4 cm, partial nephrectomy can be used to replace radical surgery. Partial nephrectomy has become the recommended surgical procedure for small renal cell carcinoma (11, 12). However, after performing the operation of retaining nephron, the patient is required to be absolutely bedridden for about 1 week, and the recovery time is long. Our center fully integrates a less traumatic laparoscopic nephron-preserving nephron surgery with the ERAS concept by promoting patient recovery and improving treatment by intervention during the perioperative period, which shows fabulous clinical application value.

From the analysis of the clinical effect indexes of the two groups, it can be seen that the recovery of intestinal function in the ERAS group is better than that in the control group, which may be related to non-enema in the ERAS group, the appropriate time of preoperative fasting and water prohibition, and the early oral feeding, which effectively promote the recovery of intestinal function. The incidence of postoperative abdominal infection and abdominal distention was caused by routine enema with soap water before renal cancer operation. Long time fasting and water prohibition may lead to the occurrence of hypoglycemia before operation. It has been found that long-term fasting and water banning easily make patients thirsty and hungry, leading to insulin resistance, which is not conducive to the recovery of patients (13). However, there was no significant difference in the time of the first postoperative exhaust between the two groups, which may be related to the individual difference between the two groups. The idea of ERAS also requires the control of intraoperative and postoperative fluid intake, so as to avoid a series of complications such as pulmonary edema and gastrointestinal edema caused by long-term massive infusion (14). In addition, we should also pay attention to intraoperative heat preservation and reduce exposure, in order to prevent stress stimulation of hormone imbalance in the body, which might lead to related complications (15). In this study, the incidence of complications in the ERAS group was lower than that in the control group, which may be related to the optimization of perioperative management in the ERAS group, such as controlling fluid input, paying attention to heat preservation during operation, early oral feeding, *etc.* Analysis of the efficiency indicators of the two groups shows that there is no statistical difference in the preoperative hospital stay between the ERAS group and the control group, while postoperative hospital stay in the ERAS group is less than that in the control group. In the case of the same discharge indication, less postoperative hospital stay in the ERAS group means that patients in the ERAS group recover faster than those in the control group. The total length of stay in the ERAS group was less than that in the control group, which can theoretically reduce the cost of hospitalization and speed up the turnover of beds. By comparing the cost of hospitalization, we found that the cost of hospitalization in the ERAS group was lower than that in the control group, which reduced the economic burden of patients and had certain economic and social benefits. In conclusion, the implementation of ERAS ensures medical safety and medical effect, improves medical efficiency and efficiency, and has high application value.

Through the evaluation of patients’ anxiety and depression before and after the operation, the results showed that the two groups of patients had the same degree of anxiety and depression before and after the operation, which was lower than that before the operation, indicating that laparoscopic nephron-sparing surgery can significantly reduce patients’ fear of the disease itself, which is conducive to psychological and mental recovery after the operation. The anxiety level of the ERAS group was lower than that of the control group, indicating that the implementation of ERAS can alleviate the anxiety level of patients. It has also been reported that the implementation of ERAS can effectively eliminate patients’ strangeness and fear of the hospital environment, reduce patients’ anxiety and tension before the operation, increase patients’ understanding of the disease, and promote patients’ dual recovery of physiology and psychology after the operation (16). The rapid development of ERAS in recent years can bring many benefits to patients, including the acceleration of postoperative rehabilitation process, the reduction of postoperative hospitalization time and cost, and the improvement of survival and prognosis. Furthermore, it was found that the overall satisfaction of patients in the ERAS group was higher than that in the control group. After 3 months of operation, the patients in the ERAS group and the control group were scored with QLQ-C30 to evaluate the impact of ERAS on long-term quality of life. The results showed that the scores of body function, role function, emotional function, and comprehensive function in the ERAS group were higher than those in the control group, but not in cognitive function, social function, and symptom dimension, indicating better life quality of the patients in the ERAS group. There was no significant difference in symptom dimension, which may be attributed to the long recovery time and the improvement of most symptoms.

Overall, our study verified that ERAS application during laparoscopic partial nephrectomy could accelerate patients’ recovery duration and enhance their prognosis as well as improve long-term life quality. It also displays certain economic advantages when compared with conventional surgical management. However, our study still has some unavoidable limitations. Firstly, our study is based on data collected in our single center (The First Affiliated Hospital of Nanjing Medical University), and some experiences might be limited or applicable only in mainland China. Secondly, many other centers abroad discharge laparoscopic or open partial nephrectomy patients in about 1–2 days, which is quite shorter than that in our routine strategies. Our perioperative period management practices are mainly based on our experiences in our center and patients’ general status, and we still need to learn from the strengths of other advanced centers.

To conclude, the results of our study demonstrated that implementation of ERAS could accelerate the recovery of patients’ physical function and effectively improve satisfaction during hospital stay and quality of life. With the growing application of the ERAS concept, the benefits achieved by patients from accelerated rehabilitation surgery will continue to increase, making the diagnosis and treatment of surgical diseases faster, better, and more efficient. With the multidisciplinary cooperation involved in using ERAS, the common cooperation as well as the management among medical personnel, their working enthusiasm, and service awareness will be significantly strengthened. This kind of improvement is also conducive to the change of traditional thinking mode and working habits. However, at present, there are still many obstacles in the implementation of the ERAS concept, but the medical benefits brought by ERAS have already been presented. The concept of ERAS is bound to achieve a wider range of promotion and application in the future.

## Data Availability Statement

All datasets generated for this study are included in the article/supplementary material.

## Ethics Statement

The studies involving human participants were reviewed and approved by Ethics Committee of The First Affiliated Hospital of Nanjing Medical University. The patients/participants provided their written informed consent to participate in this study.

## Author Contributions

ZW and MG designed the study. CM collected the patients’ clinical information. CM and AY conceived the concept of this study and helped to draft the manuscript. HY contributed to the statistical analysis. All authors approved the final manuscript.

## Conflict of Interest

The authors declare that the research was conducted in the absence of any commercial or financial relationships that could be construed as a potential conflict of interest.
